# Clinical and genetic analysis of four Chinese patients with holocarboxylase synthetase deficiency and metabolic acidosis

**DOI:** 10.1186/s13023-025-03723-2

**Published:** 2025-09-30

**Authors:** Zhenzhu Zheng, Weilin Peng, Yiming Lin, Weihua Lin, Gaoxiong Wang

**Affiliations:** 1 Neonatal Disease Screening center, Quanzhou Children’s Hospital, Quanzhou, China; 2 The School of Clinical Medicine, Fujian Medical University, Fuzhou, China; 3 Quanzhou Children’s Hospital, Quanzhou, China; 4 Department of Hepatobiliary Surgery, The Second Affiliated Hospital of Fujian Medical University, Quanzhou, China

**Keywords:** Holocarboxylase synthetase deficiency, Clinical symptoms, c.1505A > G, c.2159delT

## Abstract

**Background:**

Holocarboxylase synthetase (HLCS) deficiency is an autosomal recessive organic acidaemia. This paper aimed to describe the clinical, biochemical and molecular features of four Chinese patients with HLCS deficiency, and to research the novel mutation.

**Methods:**

Tandem mass spectrometric analysis of elevated 3-hydroxyisovaleryl carnitine (C5OH) on dried blood spots was performed. Next-generation sequencing was then used to make a definite diagnosis, and the related variants were checked in several databases.

**Results:**

The four patients exhibited varying degrees of clinical symptoms, abnormal biochemical analysis and acylcarnitine profile. A total of six mutations in the *HLCS* gene were identified, including one novel missense mutation [c.1505 A > G (p.Gln502Arg)], and one frameshift mutation [c.2159delT (p.Leu720Profs*31)]. The variation c.1505 A > G (p.Gln502Arg) is predicted to be possibly damaging by several in silico prediction programs. Another frameshift variation, c.2159delT (p.Leu720Profs*31), is classified as uncertain significance.

**Conclusions:**

A novel variation c.1505 A > G (p.Gln502Arg) expands the mutational spectrum of the *HLCS* gene. Patient 4 is the first patient diagnosed as HLCS deficiency carrying the c.2159delT (p.Leu720Profs*31) variation. The results may contribute to a better understanding of the clinical course and genetic characteristics of patients with HLCS deficiency.

## Introduction

Multiple carboxylase deficiency (MCD, MIM: 253270), a common heritable organic acidaemia, is caused by defects in either biotinidase or holocarboxylase synthetase (HLCS, EC 6.3.4.10). The enzyme HLCS plays an important role in covalent binding biotin to four biotin-dependent carboxylases in mammals, namely, pyruvate carboxylase (PC, EC 6.4.1.1), acetyl-CoA carboxylase (ACC, EC 6.4.1.2), propionyl-CoA carboxylase (PCC, EC 6.4.1.3), and 3-methylcrotonyl-CoA carboxylase (MCC, EC 6.4.1.4) [1]. HLCS deficiency is inherited in an autosomal recessive pattern. The overall incidence of HLCS deficiency worldwide is approximately 1/200,000 live births [2]. In China, the incidence is estimated to be 1/930,600 live births [3, 4]. In Japan, the disorder occurs frequently with an incidence of < 1/100,000 live births [5]. HLCS deficiency is more frequent in the Faroe Islands than in other areas, with an incidence of 1/10,000, which may be 10 times higher than the incidence in other countries [5]. Symptoms of HLCS deficiency include metabolic acidosis, skin lesions, vomiting, hypotonia, drowsiness, seizure, hyperammonaemia and developmental delay [4, 6]. Different phenotype (early or late onset) may be related to a mutation spectrum in the *HLCS* gene [7].

The pathogenic gene *HLCS*, located on chromosome 21q22.1, comprises 14 exons and 726 amino acids. The mutation spectrum in the *HLCS* gene varies greatly among different ethnic groups [7]. As reported by Yang et al., Leu237Pro and 780delG are the most common mutations in Japanese patients and account for 50% of the mutant alleles [8–10]. The IVS10 + 5G > A mutation is common and acts as a founder mutation in European patients [8, 11]. Tammachote et al. reported that all of four Thai patients carried a c.1522 C > T (p.Arg508Trp) mutation, which suggested that p.Arg508Trp was the most common mutation in Thai patients [12]. The p.Arg508Trp and c.1088T > A variations are hotspot mutations in Chinese patients with HLCS deficiency [13, 14]. The definitive diagnosis of HLCS deficiency is made according to enzyme activity or genetic analysis. More novel mutations have been reported in recent years. For example, Donti et al. have reported five patients with HLCS deficiency carrying six novel pathogenic mutations: c.500 A > C (p.Tyr167Ser), c.1532 A > T (p.Asn511Ile), c.2078G > C (p.Gly693Ala), c.977G > A (p.Gly326Glu), c.1710 C > G (p.Asn570Lys), and c.1519 + 5G > A [15]. Wu et al. have reported a 6-month-old female patient possessing a novel splice site pathogenic variant, c.2010-1G > A [16]. A novel frameshift mutation, c.1006_1007delGA (p.Glu336Thrfs*15), in a one-year-old Chinese boy was reported by our group [17].

However, information about *HLCS* mutations is still limited. Further investigation into the mutation spectrum of each ethnic group is essential for making a definitive diagnosis through DNA examination of patients with MCD. Herein, we retrospectively analysed four patients with HLCS deficiency. The clinical symptoms, acylcarnitine profiles and genetic results were studied.

## Materials and methods

This study was approved by the Ethics Committee of Quanzhou Children’s Hospital of Fujian and prepared in accordance with the Health Insurance Portability and Accountability Act (HIPAA) regulations. We retrospectively analysed four Chinese patients (two males and two females) diagnosed with HLCS deficiency. With the written informed consent of the parents, filter-paper dried blood spots (DBS) were collected and screened for several inherited metabolic diseases (IMDs) including MCD. The acylcarnitine profiles, including 3-hydroxyisovaleryl carnitine (C5OH), propionylcarnitine (C3), acetylcarnitine (C2), and free carnitine (C0), were analysed using liquid chromatography-tandem mass spectrometry (LC-MS/MS, ACQUITY TQD; Waters, Milford, MA, USA). The C5OH/C3 and C3/C2 ratios were calculated. Blood gas analysis was performed using an automated blood gas analyser (Cobas B221, Roche Diagnostics GmbH, Mannheim, Germany). All exons and adjacent noncoding regions of abnormal C5OH-related genes were enriched using polymerase chain reaction (PCR) and then screened through next-generation sequencing (NGS) using a NextSeq 500/550 Buffer Cartridge v2 Sequencing Kit on the Illumina NextSeq 500 platform.

The identified variants were checked in HGMD, ClinVar, ESP, ExAC consortium, and the 1,000 Genome Project database. The unreported variants were assessed using SIFT, PolyPhen-2, PROVEAN, and MutationTaster. Multiple amino acid sequences of different species were extracted from National Center for Biotechnology Information (NCBI) and aligned to evaluate evolutionary conservation using DNAman software. To build three-dimensional (3D) models of HLCS, homology modeling was employed using Swiss Model Workspace with PDB accession number P50747, and PDB file was then submitted to Swiss-Pdb Viewer 4.10.

## Results

### Clinical characteristics and genotypes

Patient 1, a female infant with a Cushing face, was admitted eight times with recurrent onset of cough, vomiting and shortness of breath since she was 2 years old. She was admitted to the gastroenterology department for repeated vomiting when she was five years and four months old. Capillary blood gas analysis demonstrated metabolic acidosis. She was infused with sodium bicarbonate, but the acidosis was uncorrectable. The DBS acylcarnitine profile revealed dramatic increases in the level of C5OH, C5OH/C0, and C5OH/C8. The plasma amino acid profile demonstrated substantial increases in leucine and valine levels (Table 1). The NGS results revealed that she carried compound heterozygous mutations of c.1522 C > T (p.Arg508Trp) and c.1088T > A (p.Val363Asp) (Table 1), and she was diagnosed with HLCS deficiency. However, she was transferred to another hospital, and was not followed up. Both p.Arg508Trp and p.Val363Asp are hot-spot mutations in Chinese patients with HLCS deficiency. As is reported, p.Arg508Trp, located in the C-terminal region, shows a decreased affinity for biotin but produces an elevated Km value (i.e., Km mutant) [18, 19]. Patient carrying p.Arg508Trp always presents a late-onset form and responds well to biotin therapy. Meanwhile, the p.Val363Asp mutation is located outside the biotin-binding region, and the enzyme shows normal to higher affinity for biotin but decreased Vmax [5]. Ling’s group had reported a patient homozygous for the p.Val363Asp variation presented with mild symptoms such as vomiting and erythematous dermatitis [4]. Patient 1 presented with cough and vomiting since 2 years old, which was consistent with the genotype of compound variations of p.Arg508Trp and p.Val363Asp.

**Table 1:** Clinical presentations and genotypes of four Chinese patients with HLCS deficiency

Patient No.	1	2	3	4
**Sex**	F		M	F
**Age at onset**	2 y	5 m	12 m	1 d
**Type**	L	L	L	E
**Clinical presentation**	Cough, vomit, shortness of breath, acidosis	Asthma, cough, mental fatigue, acidosis	Skin rash, tachypnea, heart failure, acidosis	Neonatal asphyxia, acidosis
**Mutation 1**	c.1522 C > T (p.Arg508Trp)	c.1522 C > T ( p.Arg508Trp)	c.1522 C > T (p.Arg508Trp)	c.2159delT ( p.Leu720Profs*31)
**Mutation 2**	c.1088T > A (p.Val363Asp)	c.1505 A > G (p.Gln502Arg)	c.1006_1007delGA (p.Glu336Thr)	c.1741G > A (p.Gly581Ser)
**Acylcarnitine and amino acid in plasma**				
**C5OH** (µmol/L, Ref: 0.07–0.5 )	5.01	5.86	3.88	1.37
**C0** (µmol/L, Ref: 8.5–50	14.39	3.64	4.86	6.5
**C5OH/C0** (Ref: 0-0.02)	0.35	1.61	0.80	0.21
**C5OH/C8** (Ref: 0.65-15)	167.0	146.5	77.6	34.25
**C3/C2** (Ref: 0.03–0.19)	0.13	0.17	0.54	0.92
**C3**/**C0** (Ref: 0.01–0.18)	0.29	0.96	0.90	1.4
**Leucine** (µmol/L, Ref: 70–270)	513.8	809.16	280.13	124.23
**Valine** (µmol/L, Ref: 67–269)	437.93	564.34	234.36	125.84

Abbreviations: F, female; M, male; d, days; m, months; y, years; E, early-onset; L, late-onset; Ref: reference

**Fig. 1:**
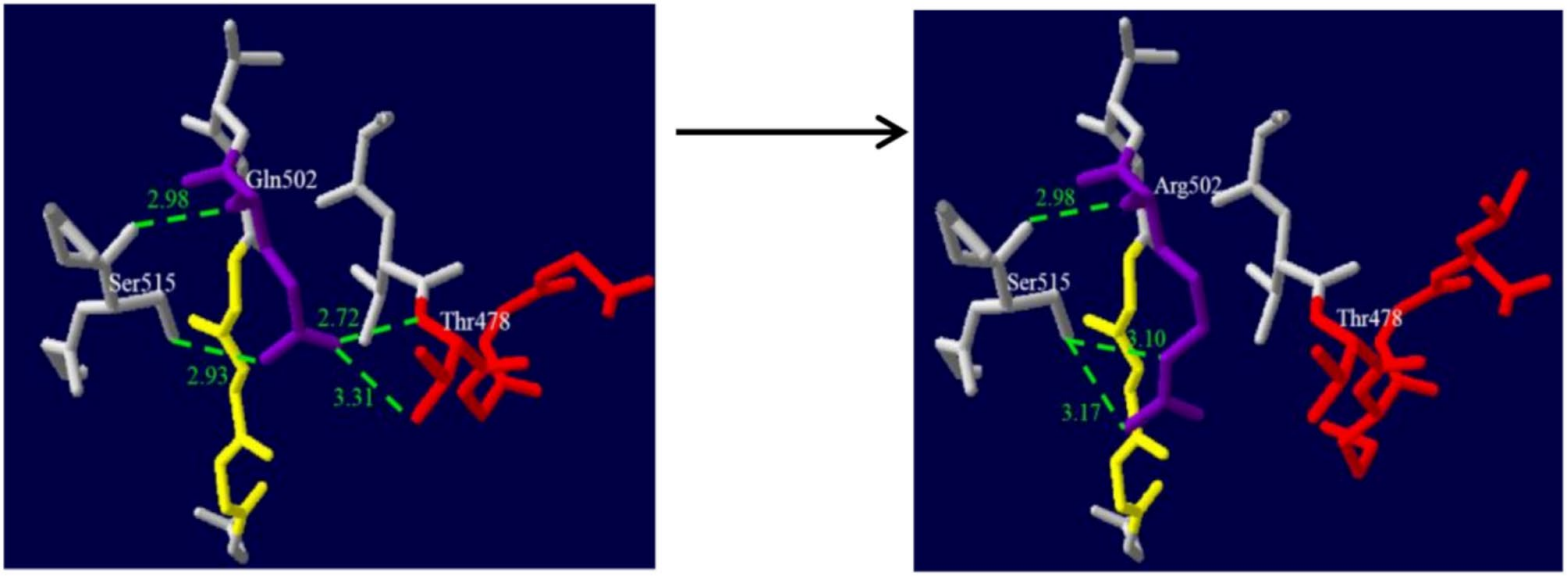
Three-dimensional models of wild-type and mutant structure of HLCS. Green dashed lines represent hydrogen bonds and green numbers show hydrogen bonds distances. **(A)** A segment of the HLCS structure shows Gln502 hydrogen bonds with Thr478 and Ser515. **(B)** A segment of the HLCS structure shows a variant c.1505 A> G (p.Gln502Arg) increases hydrogen bonds with Ser515, but removes hydrogen bonds with Thr478, which might affect the structure and function of the HLCS protein. (The color in this figure is selected by the Secondary Structure Succession of Swiss-PdbViewer 4.10.)

**Fig. 2:**
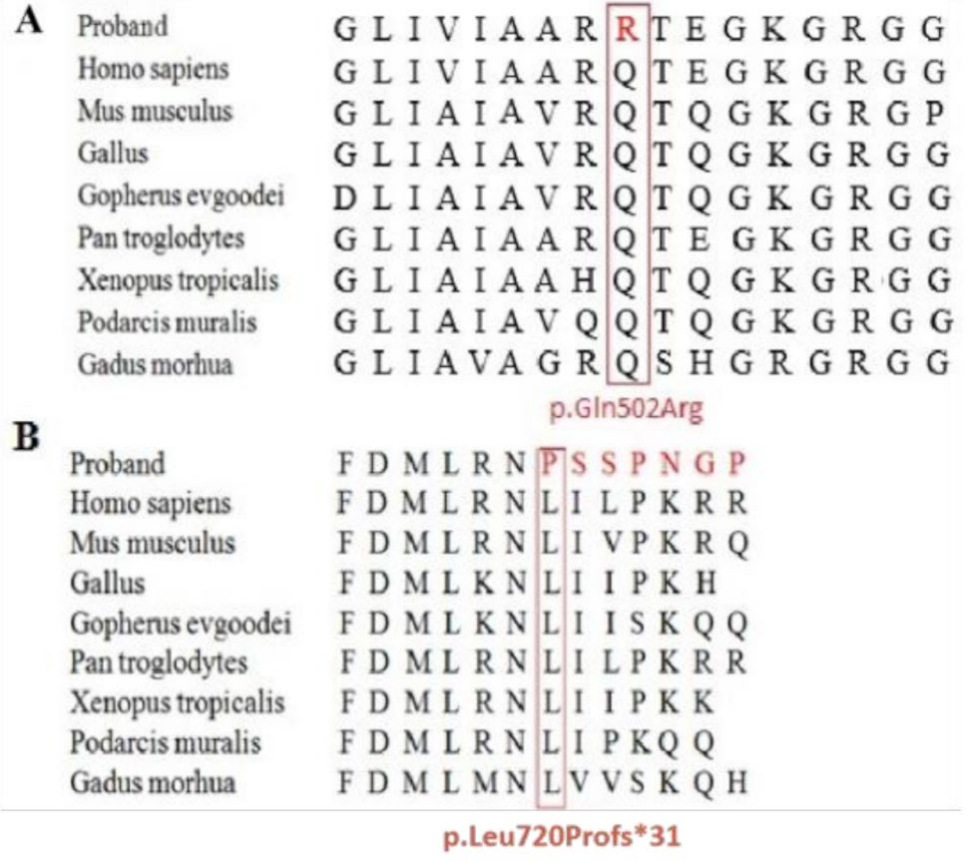
**A**. Conserved amino acid sequences of HLCS (amino acid 502, highlighted by a red box). **B**. Conserved amino acid sequences of HLCS (amino acid 720, highlighted by a red box).

Patient 2, a male patient with a fatty face, was admitted for asthma and cough at five months old. He also presented with mental fatigue, deep shortness of breath, thick breathing sounds in both lungs, and scattered wheezing sounds. The capillary blood gas analysis indicated acidosis, but the metabolic acidosis was uncorrectable. The concentration of blood glucose changed greatly between 0.08 and 21.28 mmol/L. The plasma acylcarnitine profile in the dried blood spot sample indicated a depletion of C0, and greatly increased C5OH levels as well as C5OH/C0 and C5OH/C8 ratios (Table 1). The plasma amino acid profile revealed substantial increases in leucine and valine levels. The molecular analysis results showed that he carried compound heterozygous mutations of p.Arg508Trp and a novel missense mutation, c.1505 A > G (p.Gln502Arg) in the *HLCS* gene, accompanied by a novel heterozygous mutation, c.581G > C (p.Gly194Ala), in the *PCCA* gene (Table 1). He was diagnosed with HLCS deficiency, and was transferred to another hospital without followed up. The c.1505 A > G mutation could not be found in the literature or the HGMD, ClinVar, 1000 Genomes, ExAC or ESP databases and is not detected in 100 healthy individuals. The nucleotide change c.1505 A > G induces an amino acid substitution of glutamine with arginine at position 502 (p.Gln502Arg), which is predicted to be possibly damaging by several in silico prediction programs, such as SIFT, PolyPhen-2, PROVEAN, and Mutation Taster (Table 2). Furthermore, the p.Gln502Arg variation resides in biotin-binding domain of HLCS. 3D models of the HLCS shows the variant p.Gln502Arg increases hydrogen bonds of Gln502 with Ser515, but removes hydrogen bonds of Gln502 with Thr478, which might distort the structure and affect the function of the HLCS protein (Fig. 1). The PhastCons value is 1.0 and the PhyloP score (7.842) is positive, thus the amino site is highly conserved. Furthermore, the Gln residue is assessed to be highly conserved among different species with DNAman software (Fig. 2 A). By the way, a synonymous variation c.1506G > A (p.Gln502=) is found in the ClinVar database. When all this information is taken into account, the c.1505 A > G (p.Gln502Arg) is considered to be likely pathogenic, and it expands the variant spectrum of the *HLCS* gene.

Patient 3 has been previously reported by our group [17]. He presented with a skin rash around the periorbital and perioral areas at one year old. Three weeks later, the rash extended to the limbs, neck and groin. Moreover, he manifested serious tachypnoea, moaning and heart failure, and he was admitted to the paediatric intensive care unit (PICU). Capillary blood gas analysis revealed serious metabolic acidosis, elevated lactate level, and elevated ammonia level. The plasma acylcarnitine profile demonstrated increases in C5OH levels and C5OH/C0, and C5OH/C8 ratios (Table 1). The plasma amino acid profile revealed a slightly increased leucine concentration and a normal valine concentration. The genetic analysis showed that he carried the common p.Arg508Trp mutation, a novel 2-bp deletion c.1006_1007delGA (p.Glu336Thrfs*15) in *HLCS* gene, and a novel 5-bp deletion c.638_642delAACAC (p.His213Profs*4) in *BTD* gene (Table 1). He was diagnosed with MCD and treated immediately with biotin (20 mg bid). The skin rash was eliminated and the normal acid-base balance was restored 5 days later. Presently, at age of seven years, he is doing well. The variation of p.Glu336Thrfs*15 is estimated to induce an amino acid substitution of glutamate with threonine at position 336, which resides in a conserved stretch of amino acids and results in truncated proteins lacking the conserved domains of HLCS. After position 336, a termination codon occurs at the fifteenth amino acid, which is predicted to be deleterious and likely pathogenic.

Patient 4, a female newborn baby 1 day old, was admitted for neonatal asphyxia. Prenatal colour ultrasound in her mother showed that the foetus had normal height of the left ventricle, slightly widened posterior cranial fossa, bilateral choroid plexus cysts, visible Cavum Vergae, and bilateral subependymal cysts. Patient 4 was also a small-for-gestational-age infant with a birth weight of 1750 g. She presented with sunset syndrome in both eyes, small eye fissures, a slightly low and flat nasal bridge, and a vertical line in both palms. Capillary blood gas analysis indicated that she had severe acidosis. The plasma acylcarnitine profile revealed a small decrease in C0 and large increases in C5OH, C3, as well as the C5OH/C0 and C5OH/C8 ratios (Table 1). The DNA analysis results showed that she carried compound heterozygous mutations of c.1741G > A (p.Gly581Ser) and c.2159delT (p.Leu720Profs*31) in the *HLCS* gene (Table 1). As was reported, an Italian boy possessed compound heterozygous variations of c.1741G > A and c.1648G > A (p.Val550Met) in the *HLCS* gene, who presented with an erythematous dermatitis located in the diaper and intertrigenous areas at the age of 5 months. The p.Gly581Ser substitution is mapped between two highly conserved amino acids of the biotin-binding region [18]. Furthermore, the variation c.2159delT has not been previously reported in the literature or registered in the HGMD, 1,000 Genomes and ESP databases. The MAF in the ExAC database is 0.000008 (1/118836). The c.2159delT variation is predicted to replace the last 7 amino acids with an incorrect protein sequence, resulting in an extension of the HLCS protein (p.Leu720Profs*31). The Leu residue at position 720 is assessed to be highly conserved among different species with DNAman software (Fig. 2 B). However, there are no experimental evidence illustrating its impact on protein function reported, and no clinical significance assessments submitted to ClinVar (https://www.ncbi.nlm.nih.gov/clinvar/variation/1677002/). According to the evidence outlined above, this frameshift variant is classified as uncertain significance. To our knowledge, this is the first patient affected with HLCS deficiency, who carries the variation of c.2159delT (p.Leu720Profs*31).

In this paper, four Chinese patients (two females and two males) with HLCS deficiency were retrospectively analyzed. Their clinical and molecular features as well as acylcarnitine and amino acid profiles were listed in Table 1. The four patients presented varying clinical symptoms, such as cough, vomit, shortness of breath, asthma, skin rash, heart failure and neonatal asphyxia, but all of them presented acidosis. Among them, three patients (Patients 1, 2 and 3) presented after 5 months and were classified as late-onset type. Furthermore, the four patients had substantially increased concentrations of C5OH, while the ratios of C5OH/C0, C5OH/C8 and C3/C0 were far above cut-off value. The C5OH level and its ratios on dried blood spots are widely used in newborn screening for HLCS deficiency. Moreover, C0 was substantially decreased to below the cut-off value in three patients (Patients 2, 3, and 4), which suggested the supplement of biotin as well as L-carnitine. Three patients (Patients 1, 2, and 3) presented some increases in leucine and/or valine levels in the plasma amino acid profile. The reason may be due to that the HLCS protein plays an important role in branched chain amino acid catabolism.

### Mutation analysis of the HLCS gene

In the four patients, a total of six mutations in the *HLCS* gene were identified, including one novel missense mutation [c.1505 A > G (p.Gln502Arg)], two frameshift mutation [c.1006_1007delGA (p.Glu336Thr*15), c.2159delT (p.Leu720Profs*31)], and three previously reported missense mutations [c.1088T > A (p.Val363Asp), c.1522 C > T (p.Arg508Trp), c.1741G > A (p.Gly581Ser)] (Table 2). Three patients (Patients 1, 2, and 3) all carried one allele of the p.Arg508Trp mutation, and had a late presentation. Otherwise, p.Arg508Trp, with a frequency of 37.5% (3/8), was the most common mutation in our patients, which was consistent with the report that p.Arg508Trp was hotspot mutation in Chinese patients with HLCS deficiency. In addition, the novel missense mutation c.1505 A > G (p.Gln502Arg) is predicted to be possibly deleterious by several in silico prediction programs (Table 2), and it expands the variant spectrum of the *HLCS* gene. The frameshift variation, c.2159delT (p.Leu720Profs*31), classified as uncertain significance, is first detected in a patient with HLCS deficiency.

**Table 2:** Mutations in the *HLCS* gene

Patient No.	Nucleotide change	Amino acid change	Mutation type	Comment	References
3	c.1006_1007delGA	p.Glu336Thrfs*15	Frameshift	Likely Pathogenic	Retorted
1	c.1088T > A	p.Val363Asp	Missense	Pathogenic	Retorted
2	c.1505 A > G	p.Gln502Arg	Missense	Likely Pathogenic	This study
1,2,3	c.1522 C > T	p.Arg508Trp	Missense	Pathogenic	Retorted
4	c.1741G > A	p.Gly581Ser	Missense	Pathogenic	Retorted
4	c.2159delT	p.Leu720Profs*31	Frameshift	Uncertain significance	This study
c.1505 A > G (p.Gln502Arg)					
SIFT	PolyPhen 2-HDIV	PolyPhen 2-HVAR	PROVEAN	Mutation Taster	ExAC MAF
Damaging (0.023)	Probably damaging (1.0)	Probably damaging (0.992)	Deleterious (-3.06)	Disease causing (1)	0

## Discussion

HLCS deficiency is an inherited disease of biotin metabolism characterized by variable and nonspecific symptoms. In this paper, the clinical and molecular characteristics of four Chinese patients with HLCS deficiency were demonstrated. All four Chinese patients diagnosed with MCD in our centre were HLCS deficiency, which was consistent with the report that more patients with MCD were deficient in HLCS activity in China [12, 20]. Meanwhile, the incidence of biotinidase deficiency (BTD) is much higher than that of HLCS deficiency in Western countries [12]. The symptoms of more than half of patients with HLCS deficiency occur in the newborn period. The onset time is variable and depends on the level of HLCS deficiency. In this paper, the age of onset varied from 1 day to 2 years, and three of the four patients (Patients 1, 2, and 3) presented after 5 months. The reason might be that all the three patients carried the common p.Arg508Trp mutation, which was associated with the late-onset phenotype [7]. The three patients had a late presentation but varying degrees of clinical symptoms, such as cough, vomiting, shortness of breath, asthma, skin rash and heart failure. All four patients had severe metabolic acidosis, which emphasized the importance of newborn screening [21]. Newborn screening permits early diagnosis and early treatment before the disease onset. This is helpful to prevent the life-threatening complications of HLCS deficiency, such as metabolic acidosis, seizure and hyperammonemia [4, 15].

Fortunately, newborn screening for HLCS deficiency, which is based on MS/MS spectroscopy analysis of elevated C5OH on dried blood spots, has been increasingly used in China. Herein, the four patients all had greatly increased concentrations of C5OH, as well as the ratios of C5OH/C0, C5OH/C8 and C3/C0. However, the C5OH index is a biomarker for several IMDs, including MCD (BTD and HLCS deficiency), 3-hydroxy-3-methylglutaryl-CoA lyase (HMG-CoA) deficiency, 3-methylcrotonyl-CoA carboxylase (3-MCC) deficiency, and 3-methylglutaconyl-CoA hydratase deficiency [22]. For example, Atwal’s group reported that two patients with HLCS deficiency were misdiagnosed with 3-MCC deficiency until additional testing was prompted [15]. As is reported, urinary organic acid analysis, for the qualification of 3-hydroxy-propionate and methylcitric acid, is helpful for differential diagnosis of 3-MCC deficiency and HLCS deficiency [4]. Furthermore, molecular genetic analysis is also emphasized for definitive diagnosis of HLCS deficiency.

In the four patients, six variations in the *HLCS* gene were identified, including four reported mutations [c.1088T > A (p.Val363Asp), c.1006_1007delGA (p.Glu336Thrfs*15), c.1522 C > T (p.Arg508Trp), c.1741G > A (p.Gly581Ser)], one novel missense mutation [c.1505 A > G (p.Gln502Arg)], and one frameshift mutation [c.2159delT (p.Leu720Profs*31)]. Otherwise, p.Arg508Trp was the most common mutation in our patients. As is reported, p.Arg508Trp is hotspot mutation in Chinese patients with HLCS deficiency. Moreover, c.1505 A > G (p.Gln502Arg), residing in biotin-binding domain, is predicted to be possibly damaging by SIFT, PolyPhen-2, PROVEAN, and Mutation Taster. 3D models manifest that the variation may distort the HLCS structure and affect the function of the HLCS protein. The PhastCons value is 1.0 and the PhyloP score is positive. The Gln residue is assessed to be highly conserved among different species with DNAman software. And the Gln residue at position 649 of HLCS is estimated to be highly conserved. Hence, the c.1505 A > G variant is classified as possibly deleterious and likely to be pathogenic, which expands the pathogenic variant spectrum of the *HLCS* gene.

The variation, c.2159delT (p.Leu720Profs*31), has not been previously reported in the literature or registered in the HGMD, 1,000 Genomes, or ESP databases. The ExAC MAF is estimated as 0.000008. The Leu residue at position 720 of HLCS is assessed to be highly conserved among different species. Until now, there are no experimental evidence about its impact on protein function reported, and no clinical significance assessments submitted to ClinVar. To our knowledge, Patient 4 is the first patient affected by HLCS deficiency, who carries the c.2159delT (p.Leu720Profs*31) variation.

In summary, the clinical, biochemical and molecular features of four Chinese patients were described in detail. The four patients exhibited a variety of clinical symptoms, but all presented severe metabolic acidosis, which emphasized the importance of newborn screening before disease onset. The c.1522 C > T (p.Arg508Trp) variation is hotspot mutation in Chinese patients, and it may be related with the late-onset phenotype. Moreover, the novel variation c.1505 A > G (p.Gln502Arg) is predicted to be possibly damaging by several in silico prediction programs, which expands the mutational spectrum of the *HLCS* gene. Another frameshift variation, c.2159delT (p.Leu720Profs*31), classified as uncertain significance, is first reported in Patient 4. All the results may contribute to a better understanding of the clinical course and genetic characteristics of patients with HLCS deficiency.

## Data Availability

The data generated or analyzed during this study are included in this article. The data are available from the corresponding author on reasonable request.

## Abbreviations

MCD: Multiple carboxylase deficiency
HLCS: Holocarboxylase synthetase
PC: Pyruvate carboxylase
ACC: Acetyl-CoA carboxylase
PCC: Propionyl-CoA carboxylase
MCC: 3-methylcrotonyl-CoA carboxylase
HIPAA: Health Insurance Portability and Accountability Act
DBS: Dried blood spots
IMDs: Inherited metabolic diseases
C5OH: 3-hydroxyisovaleryl carnitine
C3: propionylcarnitine
C2: acetylcarnitine
C0: free carnitine
LC-MS/MS: Liquid chromatography-tandem mass spectrometry
PCR: Polymerase chain reaction
NGS: Next-generation sequencing
NCBI: National Center for Biotechnology Information
3D: Three-dimensional
PICU: Pediatric intensive care unit
BTD: Biotinidase deficiency
HMG-CoA: 3-hydroxy-3-methylglutaryl-CoA lyase
3MCC: 3-methylcrotonyl-CoA carboxylase
